# Scientific Items

**Published:** 1878-07-20

**Authors:** 


### *Scientific Items.*

### THE TELEPHONE IN AUSCULTATION.

In reference to the statement of a correspondent of the *London Medical Journal* that with the telephone he can hear, at thirty yards, the sounds of the chest, the *Dublin Press and Circular* remarks: "The time may come when our fashionable physicians will have consulting-rooms in the large provincial towns, each having telephonic communication with his London consulting-room, where he will sit, examine and prescribe for patients who would find it inconvenient to come up to town to consult him. Of course, patients would have to attend at the provincial consulting-room at such times as he would appoint. Bristol patients could attend at 10 o'clock a. m., Birmingham at 10:30, Oxford at 11, and so on. There would probably be some difficulty about the fees; they could not be transmitted by telephone. But these could be paid to an agent or secretary." —*Drug. Circ.*

### A SINGULAR FIRE.

Not long since, the *Hartfort (Conn.) Courant* related the following instance, which illustrates the trite proverb that accidents will happen in the best regulated families. In one of the most careful households in that city, where fenders guard the fireplaces and safety matches aggravate the strange visitor, smoke was discovered in a room adjoining the one where the family were at breakfast. Investigation showed that a chair in the room was burning. How it could have taken fire was a mystery, until it was noticed that the sun's rays, falling on a large magnifying lens used to study photographs with, had been concentrated through it upon the chair, and had set it burning. If the family had not fortunately selected for breakfasting an hour when the sun is pretty near the zenith, and so prudently fixed it to have some one in the room at that dangerous time, the whole house might have been mysteriously destroyed.

---

The sunfish has repeatedly injured the submarine cable between Portugal and Brazil and along the east coast of South America. Splinters of bone have been found thrust into the cable through the several coverings so deep as to affect the electric wires. A small species of marine animal also appears to devote its special attention toward boring and destroying cables. Whales have likewise caused great damage to cables. A short time ago the cable in the Persian Gulf ceased to work. Examination was made, and it was found that a whale, which was entangled in the cable, had broken it. The animal was covered over with parasites, and in its efforts to free itself of them by rubbing its body against the cable the cable was broken, and one of the ends then coiled round the whale in such a way that it was unable to free itself, and was thus suffocated.

---

The Microphone is an apparatus which holds the same relation to sounds that the microscope does to minute and invisible objects.

Attached to the telephone, the faintest and otherwise imperceptible sounds become audible; even from the distant end of the telephone, the crawling of a fly, and even its breathing may be heard. It is supposed that the moving of sap in the capillary tubes of trees, like the murmuring ripples of a brook may be heard, and that the circulation of the blood in the minutest vessels will be so distinct that a new and interesting field will be opened up to the physiologist, and wonderful discoveries made in the diagnosis of disease.

